# Machine learning in drug delivery

**DOI:** 10.1016/j.jconrel.2024.06.045

**Published:** 2024-06-26

**Authors:** Adam J. Gormley

**Affiliations:** Department of Biomedical Engineering, Rutgers, The State University of New Jersey, Piscataway, NJ 08854, United States

**Keywords:** Machine learning, Artificial intelligence, Drug delivery, Controlled release, Formulation, Encapsulation

## Abstract

For decades, drug delivery scientists have been performing trial-and-error experimentation to manually sample parameter spaces and optimize release profiles through rational design. To enable this approach, scientists spend much of their career learning nuanced drug-material interactions that drive system behavior. In relatively simple systems, rational design criteria allow us to fine tune release profiles and enable efficacious therapies. However, as materials and drugs become increasingly sophisticated and their interactions have non-linear and compounding effects, the field is suffering the Curse of Dimensionality which prevents us from comprehending complex structure-function relationships. In the past, we have embraced this complexity by implementing high-throughput screens to increase the probability of finding ideal compositions. However, this brute force method was inefficient and led many to abandon these fishing expeditions. Fortunately, methods in data science including artificial intelligence / machine learning (AI/ML) are providing ideal analytical tools to model this complex data and ascertain quantitative structure-function relationships. In this Oration, I speak to the potential value of data science in drug delivery with particular focus on polymeric delivery systems. Here, I do not suggest that AI/ML will simply replace mechanistic understanding of complex systems. Rather, I propose that AI/ML should be yet another useful tool in the lab to navigate complex parameter spaces. The recent hype around AI/ML is breathtaking and potentially over inflated, but the value of these methods is poised to revolutionize how we perform science. Therefore, I encourage readers to consider adopting these skills and applying data science methods to their own problems. If done successfully, I believe we will all realize a paradigm shift in our approach to drug delivery.

## 1. The rise of artificial intelligence in drug delivery

**Fig. 1.**
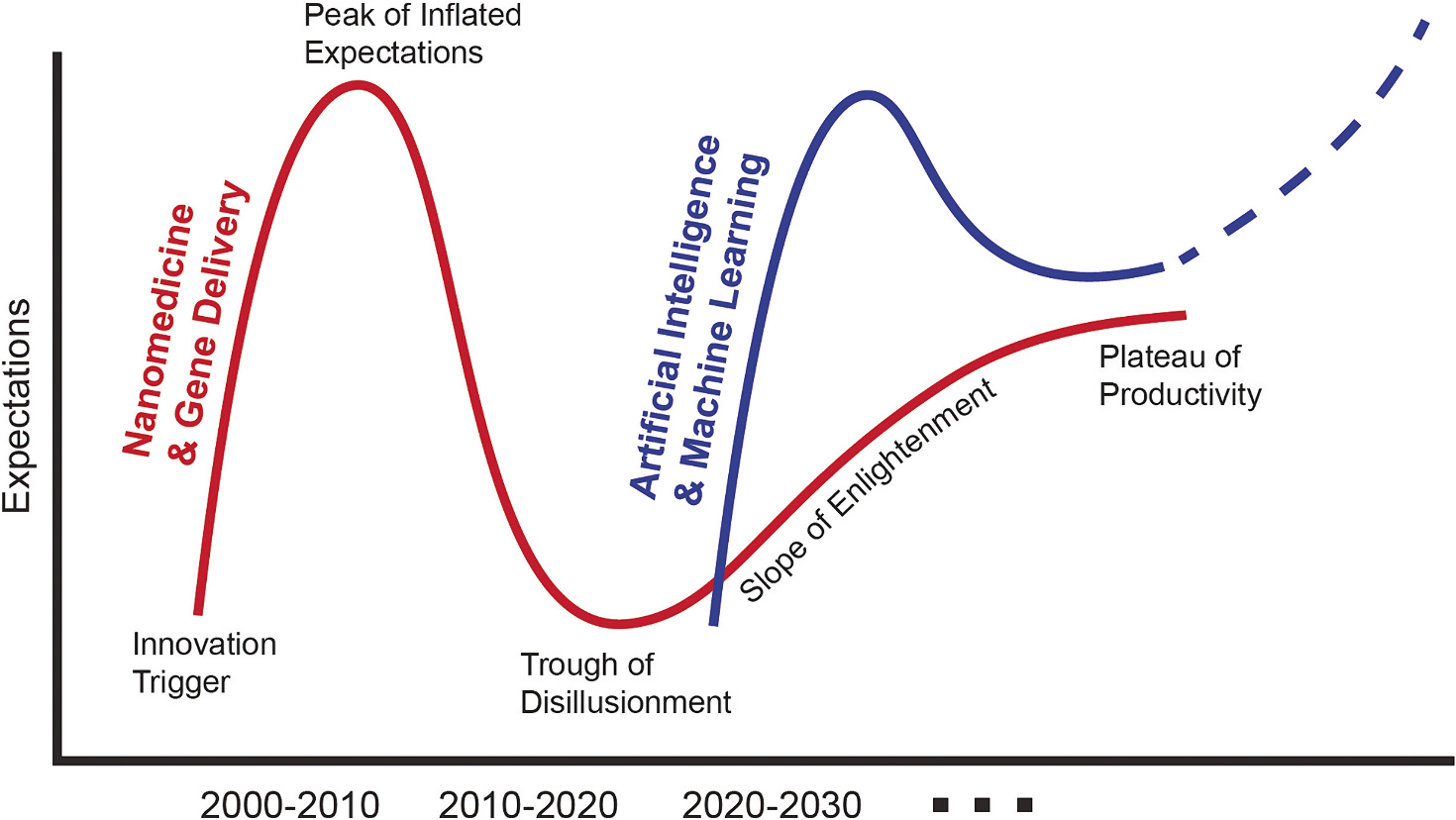
Gartner hype cycle. Dramatic innovations often follow a characteristic cycle of inflated expectations followed by troughs of disillusionment. Indeed, the field of nanomedicine and gene delivery experienced this cycle until the mRNA lipid nanoparticle vaccines demonstrated the true potential of these technologies. Now, we may be experiencing a similar cycle in artificial intelligence / machine learning (AI/ML). However, I do not think the depth of the trough will be quite so pronounced and I imagine near boundless potential for Al/ML in drug delivery in the long-term.

The recent publication of ChatGPT has suddenly brought the power of artificial intelligence (AI) to mainstream attention. Experts and media outlets are almost universally proclaiming a revolutionary future driven by AI that will touch upon every aspect of our lives. However, many rightfully ask if the hype will ultimately translate to the redefined future many are predicting. Such skepticism is valid as the Gartner hype cycle suggests that we may be within an exponential explosion of inflated expectations that is classically followed by troughs of disillusionment (Fig. 1). I remember observing these trends in the field of drug delivery during my PhD in the late-2000’s when nanomedicine and gene delivery experienced similarly inflated expectations. In hindsight, my own thesis combining gold nanorods and polymer-drug conjugates for photothermal cancer therapy seems to almost epitomize the unrealistic expectations we placed on these technologies to transform medicine. Fortunately, the field experienced a dramatic renaissance of enlightenment when mRNA lipid nanoparticle (LNP) vaccines drove immunity through the COVID-19 pandemic.

This history leads us to an important question about the current exponential rise of AI and its short- and long-term impact on drug delivery. While many scientists with long careers in drug delivery may view this as just another hype cycle, I see a much more durable future for this emerging technology. This optimism stems from my own experience using these tools to solve research problems. I do not have a training background in data science and only started playing with these tools when my lab started drowning in complex data following years of automation and high-throughput process development. My department chair suggested I try machine learning (ML) to deconstruct the data, but I was initially skeptical due to the hype around AI even in 2019. However, I will never forget the day my student trained our first model and observed its powerful ability to model complex material behavior. Subsequent studies in collaboration with Michael Webb at Princeton University correlating polymer designs to protein stabilizing behavior revealed the true potential of AI in the fields of drug delivery and biomaterials science. From these experiences, I became convinced that the future was bright for AI in materials science.

Utilization of big data is already revolutionizing how we develop and interact with smart technologies. While massive data sets may appear noisy, a deeper dive into this nuanced information can extract remarkable value. Indeed, the devices we interact with now depend on this repository of information to make predictions and increase our browsing productivity. Clearly, these techniques were developed with electronic devices in mind, but it is widely expected that big data may also be useful to a wider variety of disciplines. For example, the problem of predicating protein folding from an amino acid sequence has long been considered a grand challenge in structural biology. With this in mind, a biennial competition called Critical Assessment of Protein Structure Prediction (CASP) was launched in 1994 to encourage efforts around the protein folding problem [1]. The challenging nature of this exciting and impactful concept attracted many to participate including DeepMind, a UK based AI company now part of Alphabet Inc. Their team developed a neural network model named AlphaFold which was trained on known protein structures in the protein data bank (PDB). With this model, DeepMind participated in the 14th CASP competition and achieved remarkably accurate predictions within error of experiments [2]. As a result, many are claiming that the protein folding problem has been solved nearly 50 years after it was first introduced by Nobel Laureate Christian Anfinsen in 1972. Enter scene: intrinsically disordered proteins.

Proteins are polymers whose primary sequence ultimately determines their structure and therefore function. Materials chemistry, like biology, has grown a great appreciation for the available diversity in polymer designs. By simply substituting minor changes in monomer sequence and chemistry, we can attain new materials with remarkably unique characteristics. Over the last 50 years, we have leveraged these highly tailorable properties to create high performance materials for drug delivery. While polymer diversity enables complexity for biology and materials science, it also presents overarching challenges. Biology patiently addressed this issue by evolving new designs over billions of years. Chemists and engineers, on the other hand, are not that patient. Instead, we spend years at school training in polymer science followed by decades in industry or academia to perfect our rationally designed new materials. As a result, our most accomplished polymer scientists are also the ones with the most experience. In effect, they were trained through experience to be excellent at their craft.

Like experts, computational models can also be trained to accomplish difficult tasks. We have seen this done at the 2020 CASP14 competition, and no doubt will see this in synthetic polymer chemistry and drug delivery. However, we do not yet have a repository of information from which we can trained advanced models. As we will discuss in the proceeding sections, new advances in high-throughput automation when combined with ML may provide the needed opportunity to adequately explore vast and fruitful structure-function landscapes in drug delivery.

## 2. Machine learning introduction

AI and ML are terms that are often used interchangeably and are in fact closely related. AI is a broad concept for using a machine to mimic the cognitive functions of human intelligence. Meanwhile, ML specifically refers to models that are trained on data to predict system behavior and inform decision making. Therefore, AI is the overarching term that includes major subfields such as ML and natural language processing. Because it is accurate to refer to either AI or ML in most data science applications, my habit is to simply combine the terms into AI/ML which I will use for the remainder of this article.

The power of AI/ML to model and predict complex relationships in seemingly disorganized data provides fundamental value to all fields of science and engineering. As intelligent organisms, we take for granted how past experiences (i.e., data) have trained our cognitive ability to detect patterns and make decisions. In science, we also collect data and use that data to make predictions with degrees of certainty. This statistical analysis is taught at a very early age in our education where simple tools such as linear regression provide our first exposure to AI/ML. Therefore, most people have been using AI/ML to understand data without even knowing it! Linear regression is of course the simplest example of AI/ML where more complex functions quickly develop polynomial expressions. The challenge, however, is that variable dependencies are difficult to rationalize the moment functions develop non-linear behavior. Exponential, quadratic, and sinusoidal relationships can have simple dependencies, but may require careful study to correlate these relationships in a rational way. Therefore, most scientists develop and apply simple models to quantitatively represent information with degrees of confidence for hypothesis testing. Unfortunately, while these modeling exercises are appropriate and usually accurate, it is not always true that this furthers our general understanding of complex structure-function relationships.

The problem of understanding complex structure-function relationships is further compounded the moment we add multiple interacting variables. Students early in their training often make the mistake of testing too many conditions at once without appropriate controls and then face complex data without obvious dependencies. The truth is that dependencies do exist, we just have a hard time deconstructing data with high dimensionality. Therefore, it is better to perform multiple small experiments that learn from each other rather than perform one large experiment where all conditions are tested at once. If you are lucky, simple linear relationships exist between one dependent and one independent variable. Unfortunately, structure-function relationships are rarely that simple and subtle material properties can compound to create very complex behavior. In data science, we call this the Curse of Dimensionality.

**Fig. 2.**
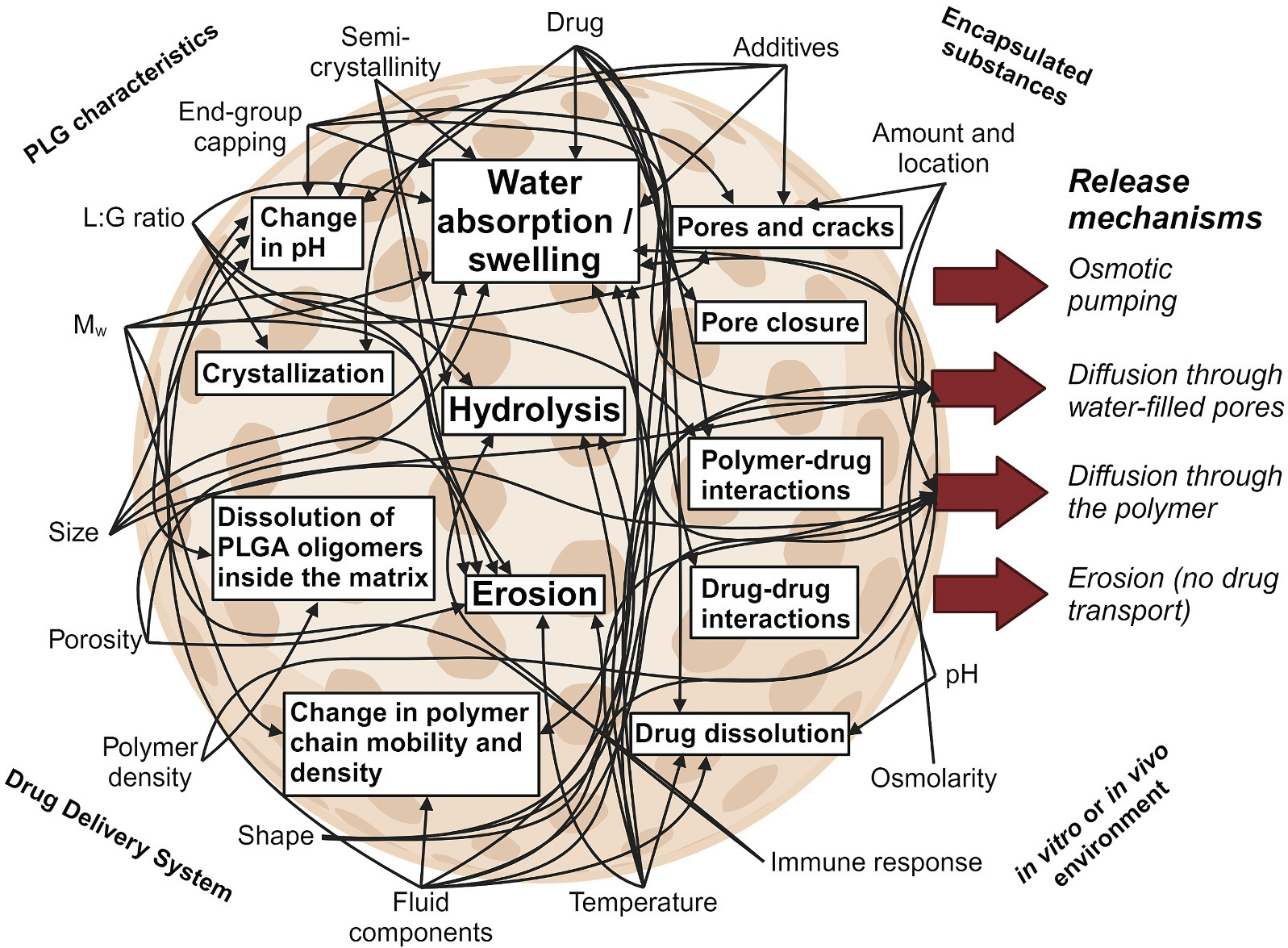
Curse of Dimensionality in drug delivery. Often, many interacting and compounding variables challenge the design problem. In this example, PLGA microparticle systems suffer the Curse of Dimensionality despite their simplified properties. Recreated with permission from Elsevier using BioRender.com [3].

The Curse of Dimensionality is particularly apparent in the field of drug delivery where many parameters can have subtle and compounding effects on drug-material interactions. In one excellent example highlighted by Axelsson et al., delivery systems made from poly(D,L-lactic-*co*-glycolic acid) (PLGA) are very common and well-studied, but suffer from many interacting parameters that complicate the design process (Fig. 2) [3]. For example, many polymer-drug material systems are characterized in a solid state to understand drug-material miscibility, drug crystallization, porosity, and more. However, the moment these systems contact water and begin to swell and hydrolyze, a cascade of interactions may dramatically change the physicochemical properties of the drug delivery system which impacts release rate. Given the complexity of these compounding interactions and the challenge associated with mapping structure-to-function as a function of time, it is no wonder that many drug delivery scientists often rely on high-throughput screening or design of experiment methods to sample parameter spaces. Unfortunately, random sampling is very inefficient and may miss key interactions that drive behavior.

Non-linear structure-function relationships with high dimensionality are best understood by AI/ML. Here, complex models are trained on known data to make accurate predictions on unseen data. If done successfully, AI/ML models can generalize even if these relationships are too complicated for humans to fully comprehend. Therefore, we do not need to rationally understand complex structure-function relationships, we just need to develop highly accurate AI/ML models to inform future designs. Neural networks, for example, are particularly configured to model problems with very high dimensionality. This power stems from their bioinspired approach to replicate our own neural connectivity which allows us to quickly weigh several competing information inputs and make approximations to inform decision making. This is why many image processing models use deep learning algorithms to generalize. However, it is likely that problems in drug delivery are not so complex that we require deep learning methods. Often, very simple supervised models such as random forests are enough to adequately model system behavior.

## 3. Data quantity, quality, and source

A common misconception is that AI/ML requires substantial data to be accurate. As a data science, it is of course true that larger quantities of data will generally produce more accurate models. However, drug delivery scientists should not be overwhelmed by the amount of data that is common in other disciplines. The reality is that our physical experiments are unlikely to scale to such an extent. However, that does not mean that AI/ML tools are not useful to the physical sciences. Quite the opposite. Learning with less is an exciting topic in data science where multiple methods may provide valuable insight from small amounts of data. Also, methods for extracting and using existing data to feed models is an exciting area of investigation.

Data mining represents one way of collecting enough useful data to train models and inform future designs. It acknowledges that decades of publicly available experiments and data are available in the literature and that these investments should not be wasted. For example, Christine Allen et al. at the University of Toronto used the COVID-19 pandemic and time at home to collect an impressive dataset for self-emulsifying drug delivery systems and long-acting injectables from existing publications [4]. As a result, they were able to train an AI/ML model on this data with high predictive capabilities [5]. However, the challenge they ran into which is true for most data mining missions is the completeness of the dataset and individual ways scientists represent information. For example, they found that many studies did not provide comprehensive documentation about their materials such as polymer structure, chemistry, molecular weight, and dispersity. In some instances, material source was not provided which prevents data miners from finding associated information on their own. Also, the representation of results is not standardized which means that the same information can be presented in very different ways. For example, some studies may use % cumulative release or % remaining, while others may use mass (ex., μg or μg/mL). These inconsistencies and the process of finding the right studies in the first place make the process of manual data mining laborious and unreliable. To combat this issue, others are developing algorithms for the automated sourcing of data from the literature. Here, keywords are used to find relevant papers and downloaded into a database. Then, these pdfs are scraped for data using image recognition to identify relevant graphs, interpret the information, and synthesize these results into consistent tabular data. No doubt, these automated methods for data mining are challenging and an area of immense opportunity to use existing information.

The problem of data inconsistency is not just a problem for the mining of data from older publications, but a problem that continues to persist. To address this challenge, members of the materials genome initiative (MGI) and beyond are highlighting the need for all investigators to publish data using FAIR (findable, accessible, interoperable, and reproducible) practices. Some journals, particularly those that publish papers with data science, now require that all raw data be provided either in the supporting information, or on publicly available databases such as GitHub. Examples of other material databases include the Materials Data Facility (MDF), Community Resource for Innovation in Polymer Technology (CRIPT), Polymer Genome, Polymer Property Predictor and Database, caNanoLab, and others [6]. For a discussion on this topic as it relates to nanomedicines, see this excellent review by Daniel Heller at Memorial Sloan Kettering Cancer Center [7]. In some of our own recent work, data has been provided as downloadable data frames which are easily accessed using a few lines of code [8,9]. The challenge is enforcement where generations of investigators are not used to organizing raw data for public disclosure. Some funding agencies are considering mandating these practices, but this requirement is not likely in the near future.

Further complicating this problem are the materials and methods used to create drug delivery technologies. Polymers are the most common material to package drugs and modulate release, but their heterogenous characteristics and limited methods for accurate characterization challenge feature representation. In contrast, AlphaFold was incredibly successful at training an AI/ML model on protein structure-function behavior because a well described repository of information was available in the PDB. Here, each protein sequence is easily represented using a variety of methods with their corresponding structure by x-ray crystallography. From this information, features that describe their structure are relatively straightforward to engineer. Unfortunately, synthetic polymers neither have such a comprehensive database nor are there obvious methods for universal representation. To tackle this problem, investigators such as Brad Olsen are creating standards for polymer representation and characterization. This includes BigSMILES, which adopts string representation of small molecules to macromolecules [10]. His lab also created PolyDat, which provides useful guidance on best practices for preparing and publishing polymer characterization data and allows for apples-to-applies comparisons [11]. Finally, he recently created CRIPT, which provides a database for these materials and their chemistries [12]. As the field of drug delivery relies heavily on synthetic polymer materials, our community needs to adopt these standardized methods so that data mining of new publications is straightforward. Also, the field of drug delivery itself needs to organize their own methods for data representation so that standard protocols are created and followed. This will likely include standard ways of representing drug release profiles and providing this data using FAIR practices. Only then will the drug delivery community maximize all new data for data mining opportunities.

**Fig. 3.**
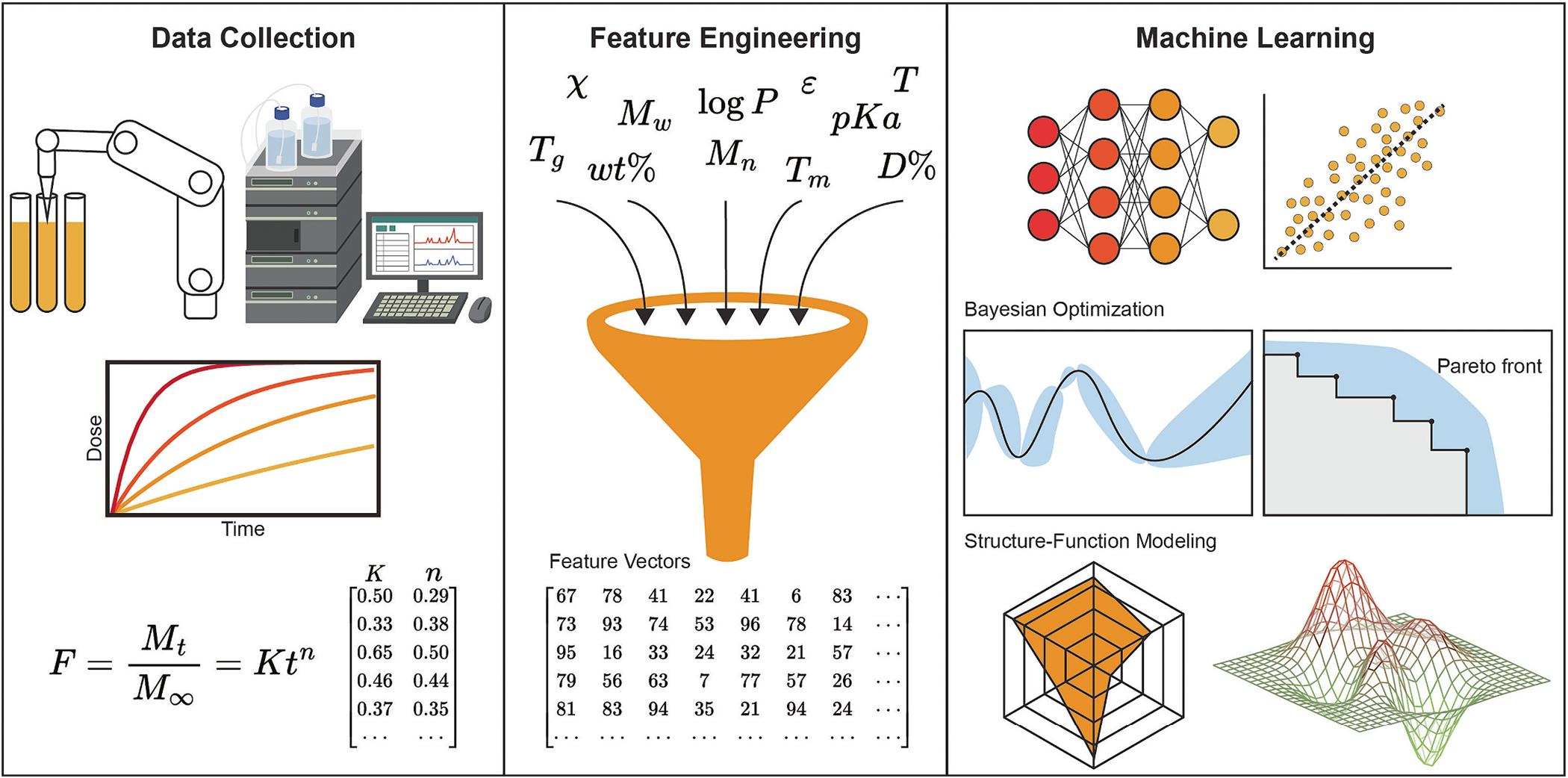
Machine learning on physical experiments in drug delivery. Methods for data curation, feature engineering, and model training drive the discovery process and enable structure-function modeling.

In most cases, the challenge of data availability is simply solved by performing new experiments (Fig. 3). However, to effectively use AI/ML, it will be important to design experiments that produce more data than traditional. In this context, convenient labware such as well plates provide the best option to enable high-throughput and combinatorial experimentation. Fortunately, many drug delivery scientists already use these experimental formats to minimize sample volume. Also, the field of drug delivery has developed these workflows over the last few decades to enable high-throughput screening experiments. These large screens solve the Curse of Dimensionality by brute force sampling and are easily reengineered to enable data science. For example, typical drug formulations labs at major pharmaceutical companies will employ a suite of liquid handling robotics and high-throughput assays to quickly generate and assess large libraries of drug-material combinations. In response, suppliers of analytical instrumentation are increasingly redesigning instruments to perform analyses in well plates and provide the raw data in downloaded formats. This trend is exciting and will greatly enable AI/ML in the physical and life sciences. However, it is important to note that the previously described challenges of data representation persist even in labs that collect their own data. Even companies that do not intend to make their data publicly available should still adopt FAIR practices for data handling to further enable and accelerate internal projects. Many companies such as Meta, Google, Apple, and others have built strong business models based on the collection and storage of useful data, and it is likely that diligent housekeeping of physical science data will provide competitive advantages as industries increasingly rely on AI/ML to accelerate discovery science.

## 4. Explainable AI to inform designs

It is exciting that so many people are embracing AI/ML tools such as ChatGPT. However, a healthy dose of skepticism is required when gaining information from AI/ML as these models are generalizing from disparate data. Just like human intelligence, AI/ML is fallible. Part of the problem is that many AI/ML models are black boxes which means that we are unable to track down source information and validate the accuracy of the results. Indeed, regular users of ChatGPT will routinely identify mistakes and hopefully put this information in context. Those that do not exercise this practice are at risk of digesting misinformation. While this problem poses great societal risk, it also poses a problem with using AI/ML to inform the design of drug delivery technologies. As scientists and engineers, we are trained to carefully plan experiments to elucidate structure-function behavior and establish design criteria. For this reason, high-throughput screens have always been appropriately characterized as fishing expeditions that do little to reveal mechanisms. As a result, high-throughput screens that became popularized in the 1990’s and 2000’s were gradually phased out for more traditional experimentation and computation that do reveal mechanisms of action. Now, with the sudden rise and embrace of AI/ML, we are at risk of repeating history.

Fortunately, not all AI/ML models are black boxes and some are highly interpretable. Often called Explainable AI, a variety of methods are being developed to specifically probe existing models and deconstruct how features are collaborating to drive system behavior. One popular example is Shapley Additive Explanation Values or SHAP which uses game theory to individually test the importance of features [13]. From this information, feature importance maps are generated including radar plots that weight their contributions. This information is very valuable and can help provide mechanistic insight. Also, quantitative structure-function maps can be generated from this information to provide detailed understandings. Often, these maps are far too complex for any human to manually generate and rationally comprehend using traditional approaches. Therefore, Explainable AI provides the right set of tools to alleviate concerns that AI/ML has the same pitfalls associated with high-throughput screens. However, since not all AI/ML models are currently interpretable including most deep learning methods, it is important to judiciously select and test models that do provide options for later interpretation. For more information, see our User’s Guide to Machine Learning [9].

While Explainable AI methods provide excellent tools to unravel the Curse of Dimensionality, they do not substitute traditional experiments to determine mechanism of action. In our recent study using AI/ML to design protein-specific polymer excipients, we used SHAP analysis to probe feature importance [8]. The results were very interesting and confirmed our hypothesis that each protein requires a specialized set of polymer material properties to achieve thermal stability above their melting temperature. However, those analyses do not reveal the mechanism of action. Therefore, we used a combination of small-angle x-ray scattering (SAXS), dynamic light scattering (DLS), circular dichroism (CD) spectroscopy, and quartz crystal microbalance (QCM) to probe protein-polymer interactions [14]. Through these careful studies, we learned that our original hypothesis was incorrect and that an unexpected mechanism of interaction was responsible for protein stabilization.

This example simply illustrates the need to still perform mechanistic studies despite the powerful outputs of these new tools. It is risky to abandon traditional characterizations as was seen during the movement towards high-throughput screening. Even with very advanced physics-informed learning, supervised AI/ML models do not accomplish rational design. They are only intended to optimize defined properties. How the AI/ML model came to a solution is only revealed through traditional mechanistic studies.

## 5. Autonomous explorations via self-driving labs

The connection between AI/ML and automation is obvious given the requirement for data with sufficient quantity and quality. As discussed earlier, high-throughput screening with the help of automation has been widely embraced in the drug delivery community for decades. Hopefully, these high-throughput experiments will be complemented with AI/ML to utilize the value of all collected data as recently done by Daniel Reker et al. now at Duke University [15]. Looking to the future, however, draws further excitement about the potential of connecting AI/ML and automation. Humans learn by continuously experimenting within their physical world to establish best practices and make decisions. Meanwhile, traditional AI/ML collects a large amount of data, trains a model, then uses this model to make predictions and decisions. In many ways, traditional AI/ML methods do not learn over time like humans do. Therefore, to make AI/ML more intelligent and better at characterizing material systems, it is better to use active learning.

**Fig. 4.**
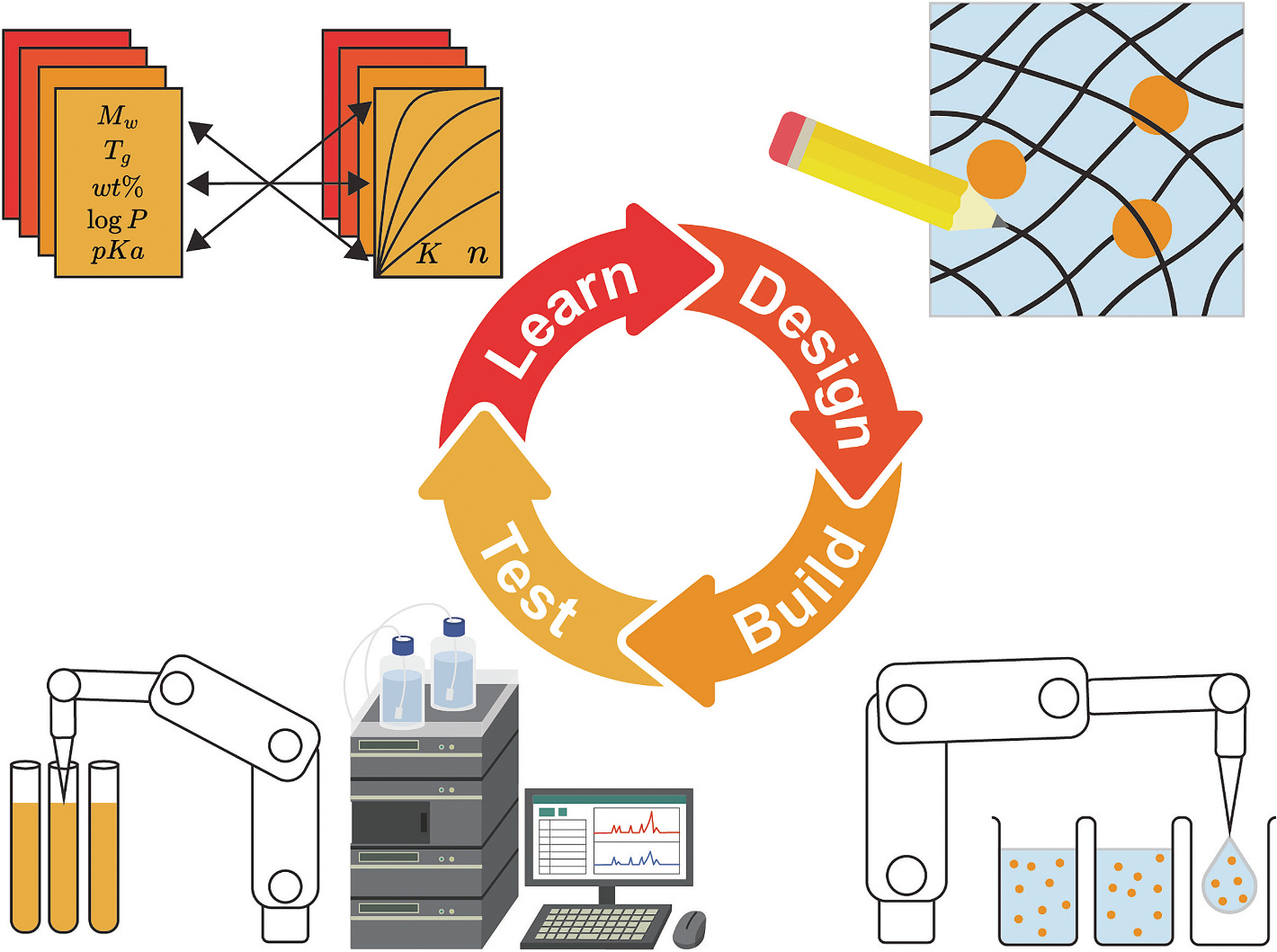
Design-Build-Test-Learn for autonomous workflows. Through the strategic integration of automation, high-throughput characterization, model training, and Bayesian optimization, active learning enables iterative experimentation and efficient learning of complex relationships.

Active learning (also known as ‘selective sampling’) utilizes a closed-loop Design-Build-Test-Learn cycle of experimentation to iteratively improve AI/ML models with new data and inform next experiments (Fig. 4) [16]. Just like human-based learning, it provides an improvable model with feedback to guide decision making. Typically, it will use Bayesian optimization to strategically map a design space by scoring the potential value of many new experiments. This acquisition function is central to active learning’s ability to efficiently map structure-function relationships with the fewest possible experiments [17]. Essentially, it is a statistical tool to design new experiments and maximize learning towards a specific outcome or set of outcomes in data scarce projects. Most importantly, active learning provides a framework for AI/ML guided decision-making and autonomous exploration of structure-function relationships.

To maximize the value and efficiency of active learning, the Build-Test portion of the workflow should be done using reliable assays with high-throughput automation. This provides the essential data quality and quantity to feed these models. As we have done in previous work, the Build-Test portion of the workflow can be performed with automation while the Learn-Design is performed offline by a human at their personal computer between each cycle. Then, new designs are uploaded to the automation for the next round of experimentation. However, the seamless integration of each portion of the Design-Build-Test-Learn workflow lends itself towards the design of a self-driving lab (SDL) [18–20]. Here, automation still performs the experiments while the Learn-Design steps are done automatically by the computer. Then, new instrumentation commands are sent to the automation to restart the workflow. While the required automation has been used for decades, autonomy for SDLs is much more difficult [21]. When achieved, systems can be programmed to autonomously attempt design optimization campaigns with unparalleled efficiency while leaving the scientist with more time to read, ask questions, and innovate. Drug delivery SDLs can also alleviate the laboratory burden of release profile timepoint sampling which is often not conducive to a reasonable work schedule (i.e., 12-h timepoints). Indeed, there is a very bright future for SDLs in drug delivery and more broadly across all disciplines. Some even believe in a Nobel Turing Challenge where a highly autonomous SDL can accomplish excellent science and win a Nobel Prize for its discoveries [22,23]. For more information about the future of SDLs in drug delivery, see two perspectives written by myself and Christine Allen [24,25].

## 6. How to get started

It is not uncommon for trainees to ask how I learned AI/ML. Afterall, I did not have any data science training prior to 2019. While younger generations are hungry to try these tools, many lack the computer science background to apply AI/ML to their own problems. Perhaps the main answer is obvious; find an AI/ML expert and collaborate. This was our approach when getting started and have worked with Michael Webb’s lab at Princeton University for the last several years. Their specialized expertise in AI/ML and feature engineering has been invaluable to many of our collaborative projects. In the meantime, our lab began to learn AI/ML on our own. To do this, the whole lab enrolled in data science courses at DataCamp (www.datacamp.com) and spent an entire summer learning to program in Python and train models. We enjoyed their interface because coursework outcomes generate XP points that we turned into a lab competition. Ultimately, this was a fun exercise that resulted in organized learning of new topics. Because of this, as well as my mandate that all data is graphed in Python, all lab members have experience in programming and AI/ML which greatly improves their job marketability. DataCamp is a paid resource, however, other free/open-source options exist including LearnPython (www.learnpython.org) and CodeCademy (www.codecademy.com), among others. Online course repositories such as Udemy (www.udemy.com) and Coursera (www.coursera.org) also have units on Python and AI/ML.

To complement these learning tools, my lab recently published *A User’s Guide to Machine Learning for Polymeric Biomaterials* [9]. Here, we describe many important concepts in AI/ML to a community with little exposure to data science in programming. Our intention with this user’s guide is to help bridge the current divide in skills and enable biomaterial and drug delivery scientists to adopt and apply these tools. In the Supporting Information, we have provided a comprehensive list of definitions for reference. Most importantly, we have provided example syntax within the publication and a Google Colab notebook so that readers can learn by example rather than theory alone. In the Colab notebook, we provided significant commentary to guide readers through each step from exploratory data analysis through explainable AI. Readers are welcome to make a copy of this notebook and import their own data to begin playing with Python and data science.

The automation of experiments is yet another technical skill that may feel prohibitive. Fortunately, many new and affordable instruments are being sold with Python application programming interfaces (API). For example, some liquid handlers like those by Opentrons can be obtained for less than $20,000 with a Python API for customized programming and remote control. The same is also true for many syringe pumps. Unfortunately, APIs are not standardized, and some older equipment use outdated RS232 serial interfaces to enable remote control. Other instrument suppliers do not offer open access which greatly limits their ability to be easily integrated. This topic was recently discussed at a Future Labs workshop at NC State where a call to action for industry to standardize APIs was discussed. Unfortunately, it is very unlikely that such standardization will be achieved soon. Therefore, automation engineers may be required for some labs looking to develop sophisticated SDLs. However, not all workflows require comprehensive remote control, and some simple systems provide excellent platforms for users to get started with automation.

## 7. Perspectives on the future

As Richard Feynman used to say, it is fun to imagine. Personally, my imagination was set fire by Richard Jones through his book ‘Soft Machines: Nanotechnology and Life’ [26]. Here, I realized that biology has taken a fascinating materials science approach to ‘machinery’ that we should be able to replicate. Obviously, we have been making proteins recombinantly for decades, but that is not what I am talking about. Instead, I am referring to mimicking biology’s machinery of proteins using synthetic materials without a DNA/RNA template or ribosomes. However, even if we did have sequence-level control of synthetic polymers, the structure-function landscape would be too impossibly large to navigate one experiment at a time. Biology solves this challenge using natural selection to guide material evolution through high dimensional parameter spaces in very high throughput. No doubt, we need to do the same. If possible, we will have the opportunity to design sophisticated nanomedicines or larger assemblies that may proactively repair or replace damaged tissue. Such imaginations have long been the focus of science fiction, but I do believe are possible if we sufficiently embrace AI/ML-guided automation to evolve nanomaterial designs. To quote Richard Feynman again, there is plenty of room at the bottom.

In the shorter term, I believe it is inevitable that AI/ML will become as common a tool as the HPLC. It is easy to disagree with this opinion, particularly those who remain skeptical by the hype. However, most people are already using AI/ML in their daily lives without realizing it. Type something into a search algorithm, and you have likely used AI/ML to find relevant content. Therefore, some seamless implementations of AI/ML in drug delivery may eventually be automatic.

The key to enabling AI/ML in drug delivery is data. Here, data can either come from experiments performed in house or from a database populated by the drug delivery community. As discussed earlier, the high-throughput generation of data is something the drug delivery community already has experience with. Therefore, the best approach is to simply apply AI/ML analyses to existing high-throughput screens. Moving forward, as individual labs gain experience, experimental workflows will naturally evolve to better feed these models and eventually embrace active learning to inform individual design campaigns. Meanwhile, the drug delivery community needs to plan for the longer-term and develop repositories of broad data such as caNanoLab that will allow us to mine information for specific projects and create AI/ML models with high generalizability like ChatGPT [7]. The creation of such a database was tried recently by Christine Allen at the University of Toronto whose results point to a bright future for collated datasets [4]. Furthermore, the community needs a dedicated focus on the appropriate feature engineering of our modeled systems. For many problems, simple descriptors representing the presence or absence of certain elements along a continuum via one-hot encoding may suffice. However, such approaches do not allow for physics-informed learning and reduce the generalizability of these models. Therefore, domain-specific descriptors, molecular fingerprints, and string- or graph-based descriptors are generally more appropriate. The menu of options is very large, which poses both a problem and opportunity for the standardization of data and how we represent this data to AI/ML models. For more information, see a recent perspective by Frank Gu et al. on this subject [27].

The discussed challenges are not meant to overwhelm, but rather point to tremendous opportunity and room for research and development. I started this Oration describing my opinion that the hype around AI/ML should be taken seriously and not disregarded as just another Gartner hype cycle. Yes, it is inevitable that some degree of sobriety will occur once the craze around AI/ML has burned out. However, I do not think that the depth of the trough of disillusionment will be as deep as that experienced with nanomedicine and gene delivery. We did see a spectacular conclusion to that story with the mRNA LNPs for COVID-19, and I expect that AI/ML will also experience a similarly productive resting place. Meanwhile, there is some concern that these tools will begin to replace a segment of the STEM workforce, where automation has notoriously displaced jobs since the industrial revolution. Let’s remember, however, that scientists are trained and employed to think, design, and solve problems, not just to do lab work. If we can outsource much of this physical work as well as the very complex challenge of correlating structure-function behavior, we will be allowed more time to perform the very human task of creative design.

We are experiencing a renaissance moment in science and technology towards AI/ML and data science. In fact, many now predict that we have entered a new paradigm of data-intensive scientific discovery [28]. To prepare our community, we need to focus on training and education as these computational tools may intimidate those with experimental backgrounds. However, science evolves, and good scientists and educators evolve with it. To do my part, my lab plans to continue publishing similarly themed *User’s Guides* to support our community [9]. Professors and educators also need to keep up with their students who are already using AI/ML tools such as ChatGPT for their education and assignments. We also need to establish FAIR practices for standardized data handling, publishing, and representation. If we can achieve these ambitious goals, the field of drug delivery will be ready for tremendous gains in productivity. Therefore, I encourage readers to try these tools and test their functional utility, being of course mindful that all techniques have pitfalls and weaknesses. Do what you do best: experiment.

## Data Availability

No data was used for the research described in the article.
